# Trends in Liver Transplantation for Acute Liver Failure in a Spanish Multicenter Cohort

**DOI:** 10.3389/ti.2025.15185

**Published:** 2025-12-16

**Authors:** Isabel Conde Amiel, Sara Martínez Delgado, Andrea Bosca Robledo, María Senosiáin Labiano, Rosa María Martín Mateos, Carolina Almohalla Álvarez, María Luisa González Diéguez, Sara Lorente Pérez, Alejandra Otero Ferreiro, María Rodríguez-Soler, José Ignacio Herrero, Laia Aceituno, Ainhoa Fernández Yunquera, Marina Berenguer, Victoria Aguilera Sancho-Tello

**Affiliations:** 1 Hospital Universitario y Politécnico La Fe, Valencia, Spain; 2 Instituto de Investigación Sanitaria La Fe, Valencia, Spain; 3 CIBERehd, Instituto Carlos III, Madrid, Spain; 4 Hospital Universitario de Cruces, Bilbao, Spain; 5 Hospital Universitario Ramón y Cajal, Instituto Ramón y Cajal de Investigación Sanitaria (IRYCIS), Universidad de Alcalá, Madrid, Spain; 6 Hospital Universitario Río Hortega, Valladolid, Spain; 7 Hospital Universitario Central de Asturias, Oviedo, Spain; 8 Hospital Clínico Universitario Lozano Blesa, Zaragoza, Spain; 9 Complejo Hospitalario Universitario A Coruña, A Coruña, Spain; 10 Hospital General Universitario de Alicante, Alicante, Spain; 11 Clínica Universidad de Navarra, Pamplona, Spain; 12 Instituto de Investigación Sanitaria de Navarra, Pamplona, Spain; 13 Hospital Universitari Vall d´Hebron, Barcelona, Spain; 14 Ajmera Transplant Centre, Toronto General Hospital, Toronto, ON, Canada; 15 Hospital General Universitario Gregorio Marañón, Madrid, Spain; 16 Universitat de València, Departamento de Medicina, Valencia, Spain

**Keywords:** liver transplantation, outcomes, acute liver failure, aetiology, sex differences

## Abstract

**Background and Aims:**

Acute liver failure (ALF) is a rare and severe condition with high mortality. Liver transplantation (LT) has improved patient outcomes. This study analysed trends in aetiology, characteristics, and outcomes of ALF patients undergoing LT in Spain.

**Methods:**

We retrospectively reviewed 217 adult ALF-LT cases from 11 Spanish centers (2001 -2020), divided into two 10-year periods. Clinical, biochemical, and outcome data were collected, and predictors of mortality were identified.

**Results:**

217 adult ALF-LT patients were included (61.8% women, mean age: 41 years). Common aetiologies were cryptogenic (26.7%), autoimmune (26.3%), and viral (18%), with sex differences. Over time, autoimmune and drug-induced liver injury increased (22.3% vs 29.8% and 13.6% vs 21.1%), with a low prevalence of acetaminophen toxicity, and hepatitis B virus declined (23.3% vs 11.4%). Despite higher infection rates (52.5% vs 66.2%) linked to stronger immunosuppression, respiratory failure (29.1% vs 16.1%), chronic kidney disease (27.1% vs 13.6%), cardiovascular events (10.6% vs 1%), and mortality (37.6% vs 17.9%) decreased. Pre-LT hypertension, pre-LT acute kidney injury, and hypernatremia at LT were independently associated with worse survival. This large multicenter study revealed temporal changes in aetiologies, immunosuppressive treatment, and post-LT complications, with an improvement in outcome.

## Graphical Abstract

**Figure:**
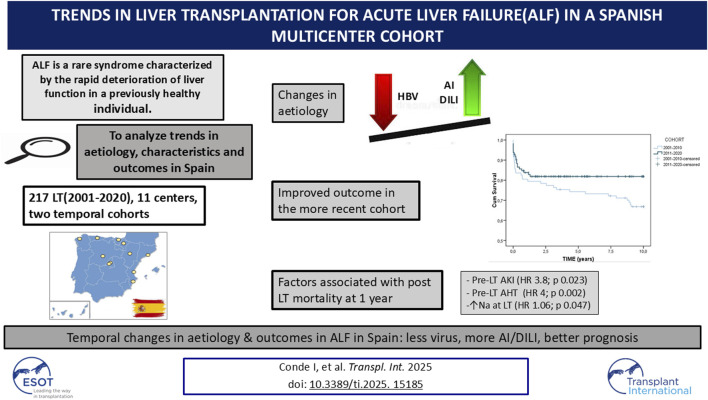

## Introduction

Acute liver failure (ALF) is a rare syndrome characterized by the rapid deterioration of liver function in a previously healthy individual. Although its prevalence is low, the exact incidence remains poorly defined. A retrospective Spanish study estimated an incidence of 1.4 cases per million population [1], while an American study reported 5.5 cases per million population [2], both published in 2007. Despite its infrequency, ALF is associated with significant morbidity and mortality, accounting for 6% of deaths related to liver disease [3].

ALF is highly heterogeneous in terms of aetiology, clinical presentation, and progression. These variations underscore the knowledge gaps in the field and the lack of large, high-quality studies. The natural history of ALF is also variable. In 10%–20% of patients, the condition is reversible, and liver regeneration occurs, leading to full recovery. However, in the remaining patients, complications such as cerebral oedema, renal failure, sepsis, and multiorgan failure are common, resulting in high mortality. The introduction of liver transplantation (LT) has significantly improved prognosis, with one-year survival rates approaching 90% in recent studies [4]. According to data from the Spanish National Transplant Organization (ONT), between 1984 and 2022, ALF accounted for 4.9% of LT indications, rising to 22% among individuals aged 16–39 years [5].

The aetiology of ALF varies depending on geographical location and age at presentation. Causes include viral hepatitis, drug overdose, idiosyncratic drug reactions, toxic ingestion, autoimmune diseases, and metabolic disorders [6, 7]. Descriptive studies have shown that in the United Kingdom and the United States, acetaminophen overdose is the most common cause [8, 9], while in highly endemic regions like India, acute hepatitis E is the leading cause [10]. In Germany, hepatotoxicity unrelated to acetaminophen was the most frequent aetiology [11]. In Spain, the most common cause of ALF was acute hepatitis due to hepatitis B virus (HBV), followed by drug and toxic substance ingestion. In more than 30% of cases, the cause of ALF could not be determined [1, 12]. Additionally, the incidence of drug-induced liver injury (DILI) has risen globally, likely due to the introduction of new pharmaceuticals, increased life expectancy, polypharmacy, and the widespread use of herbal products. DILI has become the leading cause of fulminant hepatic failure in both the United States and Europe [8, 13].

Sex-based differences have been observed in the aetiology of liver disease leading to LT, with several studies documenting sex inequities in access to LT [14, 15]. Specifically, women are at higher risk of developing DILI, and they tend to experience more severe outcomes and increased susceptibility to hepatotoxicity-related ALF [16, 17]. A recent article showed sex disparities in waitlisting and LT for ALF [18].

There are few studies on ALF in Spain, and none have been conducted in the past decade. Previous studies include a multicentre retrospective analysis of cases from 1992 to 2000 and a unicentric prospective study covering 2000–2010 [1, 12]. Despite Spain having one of the highest rates of LT *per capita*, the outcomes of LT in ALF patients have not been specifically analyzed.

This project aims to fill this gap by evaluating the recent indications, management, and outcomes of LT in ALF patients in Spain. Data were gathered from 11 large, renowned LT centers, that performed a total of over 400 LTs annually as of 2020, according to the Spanish Registry of Liver Transplantation (RETH). [5]. Our primary objectives were to describe: (i) the evolution of indications for LT in ALF, (ii) the changes over time in ALF-LT outcome, and (iii) the predictors of early post-LT mortality at 1 year. Our secondary objective was to highlight sex-based differences in ALF-LT.

## Materials and Methods

### Study Design

We conducted a retrospective Spanish multicenter study involving 11 centers with extensive experience in LT. These centers accounted for 43% of the total number of LTs performed in Spain in 2020 [5].

Urgent LT performed in patients >18 years due to ALF between 2001–2020 were included. Criteria for ALF were a severe acute liver injury lasting less than 26 weeks, with jaundice, liver synthetic failure (INR ≥1.5 or prothrombin rate <40%), and hepatic encephalopathy (HE) in a patient without known chronic liver disease.

Exclusion criteria comprised patients under 18 years old and patients with pre-existing liver disease. The acute manifestation of certain chronic liver diseases (Wilson’s disease, HBV reactivation in a non-cirrhotic liver, acute Budd-Chiari, and autoimmune hepatitis) was included as an exception. Patients with prior LT and acute liver injury due to primary graft nonfunction or other causes were excluded.

Data were acquired from each LT center through a review of medical records.

### Indications of LT in ALF and Legal Situation in Spain

In Spain, when a patient experiences ALF, the criteria to indicate an urgent LT are based on either fulfilling King`s College Criteria (KCC) [19], Clichy criteria [20] or presence of HE. A national urgent code is activated, enabling the allocation of the first suitable organ available within the country to the ALF patient. The median time until a liver is offered is approximately 40 h, and around 50% of patients receive a LT within 24 h [21].

### Ethical Statement

This study was approved by the Ethics Committee of Clinical Research of La Fe Universitari and Politécnic Hospital (ref number: 2021-096-1) and was conducted according to the standards of Good Clinical Practice, adhering to the ethical principles outlined in the 1975 Declaration of Helsinki.

An exemption from the requirement for informed consent was granted due to the retrospective nature of the study. Some patients had been relocated or were no longer reachable during the study period.

To ensure confidentiality, patient information included in the database was anonymized and identified by a numerical code, in compliance with data protection legislation.

### Collected Variables

The recorded variables included donor and recipient demographic features, epidemiological information, clinical and biochemical data before and after LT, clinical post-LT outcomes, patient and graft survival and variables associated with mortality.

- Recipient variables (demographics, co-morbidities and toxics abuse)
- Variables pre-LT associated with ALF: aetiology, type of presentation, clinical data, hepatic and extra-hepatic complications, KCC and Clichy criteria, management (antibiotic prophylaxis, N-Acetylcysteine (NAC) and Molecular Adsorbent Recirculating System (MARS)), days of admission until LT and on the waiting list (WL)
- Biochemical tests before LT (at admission, on days 3, 7, and the day of LT).
- Donor and surgical related-variables
- Histology of the explanted liver (massive or sub-massive necrosis)
- Early post-LT follow-up (1st–3rd month): days in the ICU and total hospitalization days, hepatic and extra-hepatic complications.
- Late post-LT follow-up: long term hepatic and extra-hepatic complications.
- Immunosuppression
- Outcome: re-LT and/or death, and causes.

### Operational Definitions

The diagnosis of cryptogenic ALF was reached after excluding any other aetiology through an exhaustive pre-LT differential diagnosis and the explant biopsy. Patients who received a LT in the context of an AI hepatitis fulfilled the criteria for ALF. No evidence of liver cirrhosis was found in the explants. The temporal classification of ALF (hyper acute, acute and sub-acute) was defined according to the interval between the onset of jaundice and the development of hepatic encephalopathy (published by O’Grady JG in 1993) [22].

Regarding pre- and post-LT complications, acute kidney injury (AKI) and chronic kidney disease (CKD) were established following KDIGO criteria [23, 24]. Renal replacement therapy (RRT) included both intermittent haemodialysis and continuous RRT. Infections were confirmed with positive culture or resolution after antibiotic treatment. Respiratory failure was defined as the necessity for mechanical ventilation, rather than in the context of HE. Early graft dysfunction was based on the definition proposed by Olthoff et al. [25], and acute liver allograft rejection was categorized following the Banff classification [26]. Finally, graft steatosis was assessed by biopsy.

### Statistical Analysis

A descriptive analysis was conducted for all the studied variables. Continuous variables are described as means or medians with standard deviation (SD) or quartiles 1 (Q1) and 3 (Q3) as appropriate, and qualitative variables as absolute and relative frequencies.

The normal distribution of outcome variables was confirmed using the Kolmogorov-Smirnov test. Chi-square and Fisher’s exact test were used to assess the degree of association between categorical variables, Student’s t and ANOVA model to compare quantitative variables, and non-parametric Mann-Whitney and Kruskal-Wallis tests to analyse the distribution of at least ordinal variables in 2 or more independent groups.

Graft and patient survival analyses were performed with Kaplan-Meier survival curves.

Variables associated with mortality and re-transplantation were determined using univariate and multivariate Cox regression tests and expressed by hazard ratio (HR) and 95% confidence interval (CI). The initial multivariate model included the variables with a p value < 0.10 in the univariate analysis. Variables with a p value above this threshold could be included if considered clinically relevant by the investigators.

A p-value of <0.05 was considered significant for all analyses.

Data analysis was performed using SPSS version 22.0 (IBM, Chicago, USA).

## Results

### Baseline Features and Management Before LT

A total of 217 adult patients received urgent LT due to ALF between January 2001 and December 2020. Among them, 134 were women (61.8%). The overall median age was 41 years old (IQR 32–53). Baseline clinical variables and pre-LT management are shown in Table 1, and analytical data on the LT Day in Supplementary Table 1.

**TABLE 1:** Clinical characteristics pre-LT.

Variable	N	
Age (years)	217	41 (32–53)
Sex (women)	217	134 (61.8)
Race	217	
Caucasian		181 (83.4)
Other		36 (16.6)
BMI (kg/m^2^)	152	25 (21.3–27)
AHT	216	25 (11.6)
Diabetes	216	8 (3.7)
Dyslipidaemia	216	18 (8.3)
Aetiology	217	
HBV		37 (17.1)
Other viruses		9 (5.1)
AI		57 (26.3)
DILI		38 (17.1)
Acetaminophen		9 (4.1)
Cryptogenic		58 (26.7)
Other		17 (7.9)
Clinical presentation	217	
Hyperacute		68 (31.3)
Acute		88 (40.6)
Subacute		59 (27.2)
Encephalopathy	212	
I-II		62 (29.2)
III-IV		150 (70.8)
Ascites	208	89 (42.8)
Respiratory failure (MV)	212	51 (24.1)
Infection	215	30 (14)
GI haemorrhage	216	12 (5.6)
AKI	213	83 (39)
RRT	213	47 (22.1)
Antibiotic prophylaxis	187	137 (73.3)
NAC	215	39 (18.1)
MARS	216	11 (5.1)
Time on waiting list (Days)	208	1 (1–2)
Meet KCC criteria	205	188 (91.7)
Meet clichy criteria	59	33 (55.9)
MELD - LT day	142	25 (19–29)

Data are given as median (IQR) or number (percentage).

Abbreviations: BMI, Body mass index; AHT, Arterial hypertension; HBV, Hepatitis B virus; AI, Autoimmune; DILI, Drug Induced Liver Injury; MV, Mechanical ventilation; GI, Gastrointestinal; AKI, Acute Kidney Injury; RRT, Renal Replacement Therapy; NAC, N-Acetylcysteine; MARS, Molecular Adsorbent Recirculating System; KCC, Kings College Criteria; MELD, Model for End-Stage Liver Disease; LT, Liver Transplant.

A small number of patients had concomitant diseases or toxic habits. The prevalence of arterial hypertension (AHT), diabetes and dyslipidaemia were 11.6%, 3.7% and 8.3%, respectively. Regarding toxic substances, the smoking rate was 27.8%, 15% consumed alcohol regularly and 7.5% were drug users. A concomitant autoimmune non-liver disease was present in 14.4%, and 12.1% reported a psychiatric disease.

The predominant aetiologies of ALF were cryptogenic (26.7%) and autoimmune (26.3%). Viral aetiologies accounted for less than 25%, with hepatitis B (HBV) being the most common (17.1%). Drug-induced liver injury (DILI) represented 17% of LT indications. Only 4.1% of patients who underwent LT due to DILI-ALF did so in the context of acetaminophen intake.

In terms of temporality, most cases were acute (40.6%) and hyperacute (31.3%). The most frequent complications were ascites (42.8%) and AKI (39%), while infections and haemorrhagic complications were uncommon. RRT was used in 22.1%. The median MELD (Model for end-stage Liver Disease) score on the day of LT was 25.

Before LT, antibiotic prophylaxis was widely implemented (73.3%). The use of NAC and especially MARS had little relevance in our cohort of patients.

All the patients were transplanted with a national urgent priority, resulting in a median time on the WL of only 1 day (IQR: 1–2 days).

Compliance with the KCC and Clichy criteria was 91.7% and 55.9%, respectively. Of note, only a limited number of patients (n = 59) had Factor V determination performed, especially during the early years.

### Evolution of Indications of LT in ALF

The cohort was subdivided into two 10-year periods (2001–2010 and 2011–2020). The number of ALF-LT remained stable overtime: 113 patients (3.6%) in 2001–2010 and 114 patients (3.1%) in 2011–2020.

**FIGURE 1:**
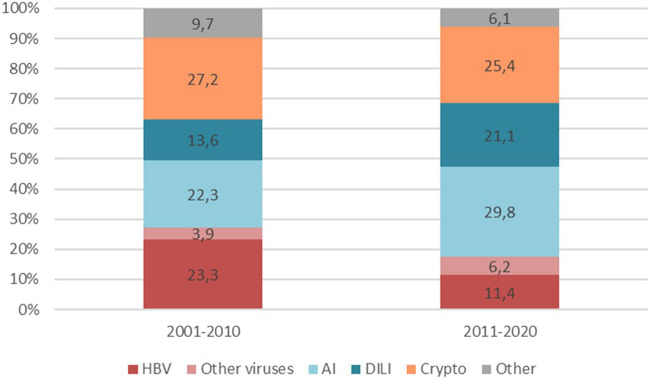
Evolution of ALF aetiologies in LT candidates Differences in ALF aetiologies between the two time periods 2001–2010 and 2011–2020 (p-value 0.115). Abbreviations: HBV, Hepatitis B virus; AI, Autoimmune; DILI, Drug Induced Liver Injury.

Cryptogenic and autoimmune were the most common aetiologies of ALF-LT overall. Autoimmune (22.3% vs. 29.8%) and DILI (13.6% vs. 21.1%) aetiologies increased with time while HBV showed a decline (23.3% vs. 11.4%), although without reaching statistical significance (p 0.115). Despite the increase in DILI, Acetaminophen toxicity was not particularly prevalent and even decreased with time (8.7% and 3.5%). Cryptogenic ALF remained stable. Other viruses, such as HAV (1% and 1.8%) or HEV (1% and 0.9%) were extremely uncommon in both periods (Figure 1).

### Changes Over Time in ALF-LT Characteristics and Outcomes

We first conducted an analysis of LT characteristics and post-LT evolution of the whole cohort (Supplementary Table 2). Most grafts were total (98.6%), with AB0 compatibility (isogroup 62.5% and compatible 37%), and showed minimal steatosis (<10% in 91.3% of grafts) and most donors were brain dead. In the early post-LT period, the main complications were infections (60.7%) and AKI (61%), while in the late post-LT period, AHT (30.3%), biliary complications (27.4%) and CKD (19.7%) predominated. The mortality rate was 27.2%, with infections (41.5%) and liver-related complications (20.8%) being the leading causes of death. The survival rates at 1, 5, and 10 years were 82%, 78% and 72%, respectively (Supplementary Figure 1).

Regarding differences over time in pre-LT characteristics and management (Table 2), there was a trend towards an increase of women (55.3% vs. 67.3%) approaching statistical significance (p 0.065) and a decline in the rate of Caucasian race (90.3% vs. 77.2%; p 0.001). Alcohol consumption was reported less frequently in recent years (21.6% vs. 8.9%; p 0.01). Antibiotic prophylaxis and, notably, the use of NAC significantly increased (65.4% vs. 78.9%; p 0.04% and 4% vs. 30.1%; p < 0.001).

**TABLE 2:** Differences over time in ALF-LT.

Variable	2001–2010		2011–2020		p-value
	n = 103		n = 114		
	n	n					
Clinical characteristics and management pre-LT					
Sex (women)	103	57 (55.3)	114	77 (67.5)	0.065
Race	103		114		**0.001**
Caucasian		93 (90.3)		68 (77.2)	
Other		10 (9.7)		26 (22.8)	
Alcohol	102	22 (21.6)	112	10 (8.9)	**0.010**
Aetiology	103		114		0.115
HBV		24 (23.3)		13 (11.4)	
Other viruses		4 (3.9)		8 (6.2)	
AI		23 (22.3)		34 (29.8)	
DILI		14 (13.6)		24 (21.1)	
Acetaminophen		5 (8.7)		4 (3.5)	
Cryptogenic		28 (27.2)		29 (25.4)	
Other		10 (9.7)		7 (6.1)	
Antibiotic prophylaxis	78	51 (65.4)	109	86 (78.9)	**0.040**
NAC	102	5 (4.0)	113	34 (30.1)	**<0.001**
MARS	102	8 (7.8)	114	3 (2.6)	0.082
Donor					
Steatosis	82		80		**0.037**
<10%		78 (95.1)		70 (87.5)	
10%–30%		2 (2.4)		9 (11.3)	
>30%		2 (2.4)		1 (1.3)	
Immunosuppression					
Induction IS	97		110		**<0.001**
Triple IS		60 (61.9)		87 (79.1)	
Double IS		33 (34)		12 (10.9)	
Other		4 (4.1)		11 (10)	
Basiliximab	82	28 (34.1)	107	61 (67)	**0.002**
Early post-LT complications					
Resuscitation unit (days)	99	6 (4–11)	113	5 (3–9)	0.077
Infection	101	53 (52.5)	110	75 (68.2)	**0.020**
Respiratory insufficiency	103	30 (29.1)	112	18 (16.1)	**0.022**
Late post-LT complications					
CKD	85	23 (27.1)	103	14 (13.6)	**0.021**
CV event	85	9 (10.6)	103	1 (1)	**0.003**
Death	101	38 (37.6)	112	20 (17.9)	**0.001**
Death 1yr		19 (19)		18 (16.1)	**0.575**

Data are given as median (IQR) or number (percentage). The bold values indicate variables that are statistically significant (p < 0.05).

Abbreviations: HBV, Hepatitis B virus; AI, Autoimmune; DILI, Drug Induced Liver Injury; NAC, N-Acetylcysteine; MARS, Molecular Adsorbent Recirculating System; IS, Immunosuppression; CKD, Chronic Kidney Disease; CV, Cardiovascular.

**FIGURE 2:**
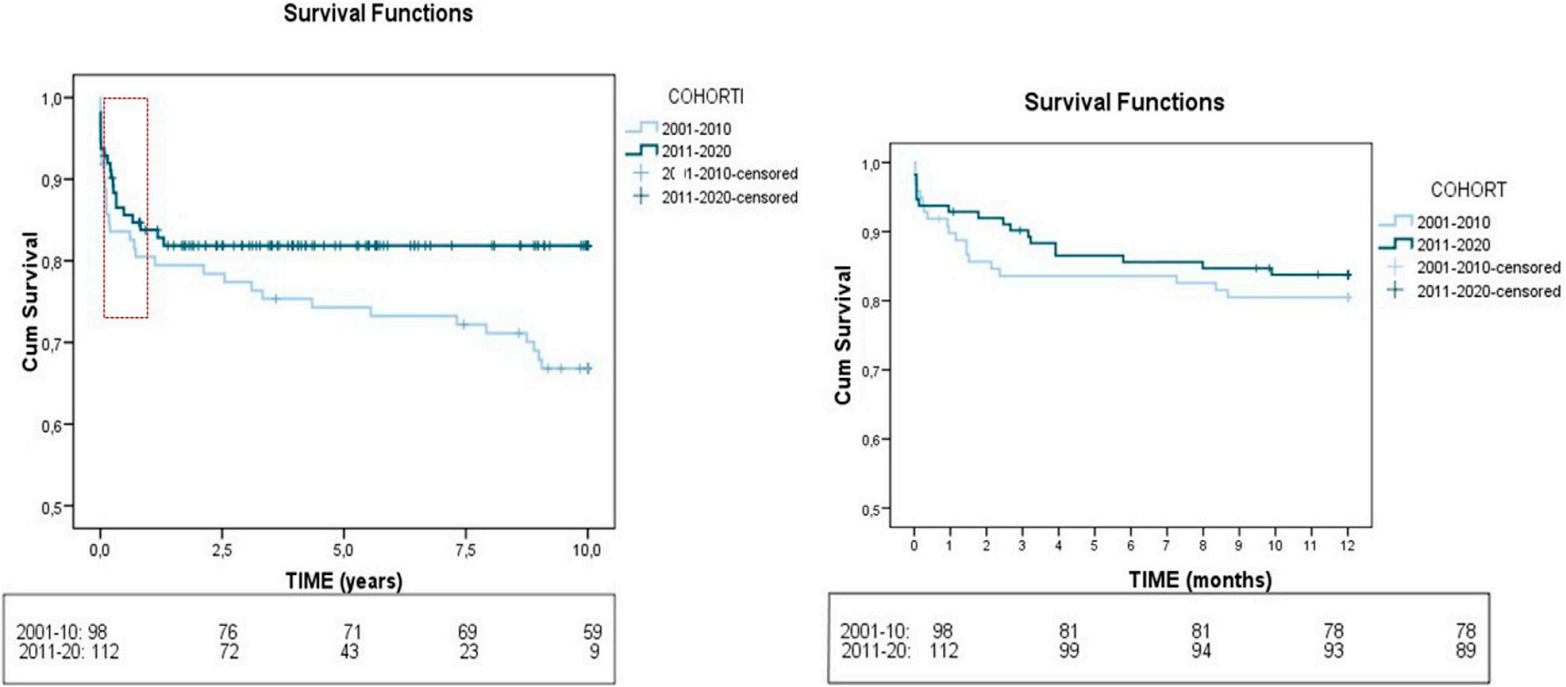
Evolution of patient survival rates between the two time periods 2001–2010 and 2011–2020. The Kaplan-Meier plot illustrates the differences in post-LT survival rates between the 103 ALF patients who underwent LT in 2001–2010 versus 114 patients who underwent LT in 2011–2020.

We also examined the changes in post-transplant management and outcome between the first and second decade (Table 2). There was a higher use of triple immunosuppression (61.9%–79.1%; p < 0.001) and basiliximab (34.1%–67%; p 0.002) in the recent cohort. Some differences were found in post-LT complications. Infection rates increased overtime (52.5% vs. 66.2%; p 0.02) while respiratory insufficiency decreased (29.1% vs. 16.1%; p 0.022). In the long-term, there was a reduction in CKD (27.1% vs. 13.6%; p 0.022), cardiovascular events (10.6% vs. 1%; p 0.003) and mortality (37.6% vs. 17.9%; p 0.001) in recent years. One year mortality improved with time, not reaching statistical significance (19% in the first cohort vs. 16.1% in the latter, p 0.575). Evolution of patient survival rates between the two time periods is shown in Figure 2. (80.5%, 74% and 67% vs 84%, 82% and 82% at 1, 5, and 10 years, respectively).

Given the observed increase in AI/DILI aetiologies, we implemented an analysis to determine whether there were differences in management and outcome when comparing AI/DILI ALF group to the rest of aetiologies (Supplementary Table 4). A total of 95 patients were transplanted in the context of AI or DILI ALF, and 122 patients had other ALF aetiologies. Subacute presentations were more prevalent in AI/DILI aetiologies (34.7% vs. 21.7%; p 0.044), with a different trend for hyperacute presentations. AKI was significantly less common AI/DILI subgroup (30.1% vs. 45.8%; p 0.024). In terms of post-LT outcome, differences were observed in the IS management (higher use of triple IS, p 0.034) and in early complications (lower requirement for RRT, p 0.015; higher incidence of infections, with a lower rate of bacterial infections, p 0.017; and a decrease in bleeding and CV complications, p 0.020 and p 0.021). A significant finding in late post-LT outcome was the lower rate of *de novo* tumours in AI/DILI aetiologies (3.6% vs. 10.6%, p 0.034), as well as lower mortality at 1-year post-LT (12% vs. 21.7%, approaching statistical significance: p 0.065).

### Factors Associated With Post-LT Mortality

Given that most deaths occurred early post-LT, we determined variables independently associated with mortality at 1-year post-LT (Table 3).

**TABLE 3:** Factors associated with post-LT mortality.

Variable	N	Univariate analysis				Multivariate analysis			
		HR	95% CI		p-value	HR	95% CI		p-value
Aetiology AI-DILI	217	0.52	0.26	1.06	0.072				
Obesity	152	3.33	1.07	10.3	**0.037**	2.693	0.972	7.456	0.057
AHT	216	2.75	1.30	5.84	**0.008**	4.002	1.222	13.106	**0.022**
Dyslipidaemia	216	2.75	1.21	6.26	**0.016**				
Acute presentation	217	0.37	0.17	0.84	**0.017**				
AKI pre-LT	213	2.83	1.46	5.5	**0.002**	3.819	1.199	12.159	**0.023**
Respiratory insuff.	212	1.97	1.01	3.82	**0.046**				
Infections	215	2.41	1.16	4.97	**0.018**	3.120	0.872	11.162	0.080
Vasopressors	150	2.14	1.02	4.5	**0.044**				
Na - LT day	189	1.087	1.027	1.149	**0.004**	1.065	1.001	1.133	**0.047**
Cr - LT day	193	1.253	1.013	1.550	**0.038**				
P - LT day	76	1.307	1.044	1.636	**0.019**				
Ammonium - LT day	70	1.007	1.001	1.013	**0.021**				
Lactate - LT day	69	1.119	1.008	1.242	**0.036**				
Factor V - LT day	37	0.893	0.803	0.993	**0.037**				

Univariate and multivariate analysis. Results of Cox multiple regression models, adjusted hazard ratio (HR), 95%CI and p-value. The bold values indicate variables that are statistically significant (p < 0.05).

Abbreviations: AI, Autoimmune; DILI, Drug Induced Liver Injury; AHT, Arterial Hypertension; LT, Liver Transplant; AKI, Acute Kidney Injury; Na, Sodium; Cr, Creatinine; P, Phosphorus.

Significant variables related to patient’s baseline characteristics, clinical presentation, pre-LT complications and laboratory data at LT predicted poor outcomes in the univariate logistic analysis. Obese patients were at a significantly higher risk of death than those with normal BMI (HR = 3.33; p = 0.037). AHT and dyslipidaemia significantly influenced survival time (HR = 2.75; p = 0.008 and HR = 2.75; p = 0.016). Acute presentation was related to lower mortality (HR = 0.37; p = 0.017). Among the complications detected prior to transplantation, AKI, respiratory insufficiency, infections and vasopressor use significantly worsened the prognosis (p < 0.05). Finally, an increase in creatinine, sodium, phosphorus, ammonium, and lactate levels and a decrease in Factor V were independently associated with death (p < 0.05). The remaining pre-LT variables were not statistically significant.

Multivariable logistic regression analysis was performed on selected baseline variables from the univariate analyses, including independent predictors with clinical relevance, that were previously identified as significant (p < 0.05) and that were available in a relevant number of patients. Obesity, AHT, AKI, infections and sodium level on LT-day were entered into the multivariable model, and AHT, AKI and sodium remained as independent risk factors (HR 4.002 p 0.022, HR 3.819 p 0.023 and HR 1.065 p 0.047, respectively).

### Differences According to Sex in ALF-LT

There was a trend towards an increase of women over time (55.3% vs. 67.5%), although this difference was not statistically significant (p 0.065) (Table 2).

Autoimmune and cryptogenic aetiologies were more frequent in women (31% vs. 19% and 31% vs. 20%) while HBV was more common in men (29% vs. 10%) (p 0.007).

Before LT, men had a higher history of alcohol, tobacco and drug consumption (p < 0.05). AKI was more frequently observed in men (52% vs. 29%). Renal function, ALT levels, platelets count and MELD score pre-LT were worse in men (p < 0.05). No significant differences were found in other pre-LT characteristics.

Early post-LT complications such as AKI (73% vs. 53%) and haemorrhage (26% vs. 14%) were more frequent in men, while rejection was more common in women (11% vs. 22.5%) (p < 0.05). Later complications including AHT (36% vs. 27%), dyslipidaemia (25% vs. 11%), CKD (24% vs. 17%) and biliary complications (32% vs. 21%) were all more frequent in men but without reaching statistical significance. Causes of death, survival and re-LT were similar in both groups (Supplementary Table 3).

## Discussion

This study includes a large multicenter cohort of patients, allowing for an accurate overview of the evolution and outcomes of LT for ALF in Spain from 2001 to 2020. The only prior Spanish multicenter study, published in 2007, evaluated ALF patients between 1992 and 2000 [1]. Our analysis covers a more recent period (2001–2020), and provides an update on the evolution of this condition in patients who eventually required LT. Additionally, there are two older European studies: a German multicentre study that included ALF patients diagnosed in 2008–2009 [11], and a second study that assessed patients included in the European Liver Transplant Registry (ELTR) database between 1988 and 2009 [27]. Although there are discrepancies in ALF epidemiology and management across Europe, our study may serve as a current benchmark for the region.

Our study highlights differences in the aetiology of ALF compared to other regions. The most frequent aetiologies in our cohort were cryptogenic, autoimmune, and viral, with a notable shift towards autoimmune and DILI aetiologies and a decreased relevance of HBV in recent years. While DILI is the most common cause in Anglo-Saxon countries, viral hepatitis remains significant in developing countries. Notably, DILI was less common in our study compared to Western countries, similar to previous Spanish data published by Escorsell in 2007 [1], but its frequency has increased over time. Another significant distinction is that acetaminophen toxicity was uncommon in our cohort and has even decreased in recent years, probably due to the implementation of NAC protocols and the fact that it is less accessible to the general population than in other countries. International cohort studies, such as one from the US, have reported similar trends, with an increase in autoimmune cases and a decrease in HBV and DILI over time [24, 28].

Some changes in the outcome of ALF-LT in recent years have been documented in our cohort. We have observed a decrease in certain short- and long-term post-LT complications: respiratory insufficiency, CKD, cardiovascular events and even mortality. The higher use of monoclonal antibodies in the induction IS facilitates the reduction of the CNI dose from the moment of transplantation, and possibly justifies the downward trend in CKD. Survival rates were consistent with data from other series reaching 82%, 78% and 72% at 1, 5 and 10 years after LT, respectively. Recent studies have reported lower mortality in recent years [1, 4, 25], with improved peri-transplant management in intensive care units being a key factor. In our study, we also detected a trend toward a decrease in 1-year post-LT mortality. However, mortality remains high in the early post-transplant period, especially during the first 3 months (13%). One notable finding is the increase in infections in the early post-LT period (although without impact on survival), which may be explained by the use of more potent immunosuppression regimes in recent years.

When compared to other aetiologies, distinct clinical characteristics were observed in the AI-DILI group. Notably, the subacute presentation was more frequent, likely associated with the early use of corticosteroids. Post-transplant, patients with AI-DILI were more frequently maintained on a triple IS regimen, and fungal and viral infections were more commonly observed. These findings may be related to pre-transplant immunosuppressive therapy, including corticosteroids, administered in an attempt to avoid LT. A significant finding was the lower rate of *de novo* tumours, despite the higher IS, and mortality in AI-DILI aetiologies. This may be related to the higher prevalence of women in this subgroup, the lower rate of toxic habits among them, and possibly the shorter follow up of this group of patients.

Several pre-transplant parameters were associated with 1-year mortality. The significant predictors of post-transplant survival in the univariate were baseline features such as obesity, AHT and dyslipidaemia, pre-transplant clinical complications (AKI, respiratory insufficiency, infections and vasopressors need), and laboratory variables (sodium, creatinine, phosphorus, ammonium, lactate and factor V). These variables are consistent with previously published prognostic factors linked to poor survival in ALF [29–31]. Serum sodium levels showed an inverse relationship with post-LT survival. Classically studies linked pretransplant hyponatremia with increased post-LT mortality, recent large-scale analyses have suggested that hypernatremia is associated with worse outcome in ALF [32]. Other variables reported in previous studies, such as recipient and donor age, ABO incompatibility and intracranial pressure (ICP) monitoring pre-LT [4, 27] did not reach statistical significance in our analysis. The use of high-quality donors (young, compatible, with minimal steatosis) may explain some of these results. We have no data on ICP; however, HE, and more specifically grade IV HE, was not statistically significant in the univariate analysis.

Regarding potential sex differences, we observed an increasing rate of women undergoing LT for ALF across years and a higher number of ALF due to autoimmune hepatitis and cryptogenic liver disease. The increasing prevalence of autoimmune diseases among women may explain this [33]. Men presented in worse clinical condition at the time of LT, leading to a higher rate of post-LT complications, except for rejection, which was more common in women. Long-term outcomes, however, were similar for both sexes, with no differences in mortality. This data is particularly noteworthy in contrast to previous series which showed sex differences in pre-LT disease course in favour of men [18, 34].

Some limitations of the study should be mentioned. The retrospective design of the study may have led to partial loss of information, especially in the early years. Inclusion of only 11 out of 26 national LT centers may potentially bias some of the results; yet we incorporated the larger centers with more expertise. Potential heterogeneity in ALF management and transplantation protocols across centers may result in biases in patient selection and therapeutic decisions. For example, antibiotics, NAC or MARS are not addressed in national protocols. In our centers, NAC was administered in all instances of acetaminophen-induced ALF. In recent years, it was also used in select cases of non-acetaminophen ALF during the early stages of HE, in accordance with published potential benefit in this clinical scenario [35]. Antibiotic prophylaxis was not universally implemented, but was consistently prescribed at the slightest suspicion of infection following clinical practice guidelines [36]. The use of MARS was minimal, probably due to the low availability of this technique in our country, the short waiting time, and the lack of evidence supporting its efficacy [37]. Finally, data from ALF patients who have not undergone LT are not available in the majority of centers. The lack of these patients may introduce a selection bias. We plan to perform a prospective study to assess this very relevant piece of information to understand the process of patient referral and LT selection.

In conclusion, with data based on 11 large reference LT centers, this study is a picture of LT for ALF in Spain and reflects the trends over time in the last 20 years. The study revealed temporal changes in aetiologies (with an increase in autoimmune and DILI aetiologies, with a marginal relevance of acetaminophen overdose, and a decreased relevance of HBV in recent years), pre-LT management, immunosuppressive treatment, and post-LT complications. Overall, outcomes in this critically ill patient group have improved with increased survival over time. Early post-LT mortality was associated with pre-transplant AHT, AKI and hypernatremia.

## Data Availability

The original contributions presented in the study are included in the article/Supplementary Material, further inquiries can be directed to the corresponding author.

## Glossary

AHT: Arterial Hypertension
AI: autoimmune
AKI: Acute Kidney Injury
RRT: Renal Replacement Therapy
ALF: Acute Liver Failure
ALT: Alanine Aminotransferase
AP: Alkaline Phosphatase
AST: Aspartate Aminotransferase
AZA: Azathioprine
CKD: Chronic Kidney Disease
CNI: Calcineurin Inhibitor
CV: Cardiovascular
DILI: Drug Induced Liver Injury
GFR: Glomerular Filtration Rate
HAV: Hepatitis A Virus
HBV: Hepatitis B Virus
HE: Hepatic Encephalopathy
HEV: Hepatitis E Virus
HR: Hazard Ratio
ICU: Intensive Care Unit
IQR: Interquartile Range
INR: International Normalized Ratio
IS: Immunosuppression
KCC: King’s College Criteria
LT: Liver Transplantation
MARS: Molecular Adsorbent Recirculating System
WL: Waiting list
MELD: Model for End-Stage Liver Disease
MMF: Mycophenolate Mofetil
NAC: N-Acetylcysteine
PDN: Prednisone
SD: Standard Deviation
