# Supplementary material for: Trends in Liver Transplantation for Acute Liver Failure in a Spanish Multicenter Cohort
Source: Transpl Int. 2025 Dec 16;38:15185. doi: 10.3389/ti.2025.15185 (PMC12752109; doi:10.3389/ti.2025.15185)
Supplement: Supplementary file 1 [file Supplementaryfile1.docx]

**Supplementary tables:**

Supplementary Table 1: Analytical data on the LT Day

| Analytical parameter | Median (IQR) |
| --- | --- |
| - Glucose (mg/dL) | 114 (93-142) |
| - Creatinine (mg/dL) | 0.8 (0.56-1.4) |
| - eGFR (ml/min) | 90 (47-100) |
| - Bilirubin (mg/dL) | 20.6 (12.1-27.9) |
| - AST (UI/L) | 605 (304-1474) |
| - ALT (UI/L) | 900.5 (364-2092) |
| - AP (UI/L) | 141 (105-218) |
| - Albumin (g/L) | 2.8 (2.5-3.1) |
| - Sodium (mEq/L) | 139 (135-142) |
| - Haemoglobin (g/dL) | 11 (9.7-12.5) |
| - White blood cells (x10^3^ /µL) | 9100 (6300-13600) |
| - Platelets (x103 /µL) | 126000 (79000-194000) |
| - INR | 3.65 (2.46-4.97) |
| - Factor V (%) | 23.3 (12-35) |
| - pH | 7.43 (7.37-7.49) |
| - Lactate (mg/dL) | 3.3 (2.3-5.9) |
| - Ammonia (μMol/L) | 158 (99-225) |

eGFR: estimated Glomerular Filtration Rate, AST: Aspartate Aminotransferase, ALT: Alanine Aminotransferase, AP: alkaline phosphatase, INR: International Normalized Ratio, MELD: Model for End-Stage Liver Disease, IQR: interquartile range

Supplementary Table 2: LT characteristics and post-LT evolution

| Post-LT characteristics | N |  |
| --- | --- | --- |
| Donor and surgical characteristics | | |
| Donor gender: women | 178 | 81 (45.5) |
| Donor age | 184 | 57 (46-68) |
| Type of LT | 215 |  |
| - Total |  | 212 (98.6) |
| - Split |  | 3 (1.4) |
| ABO/Rh compatibility | 208 |  |
| - Isogroup |  | 130 (62.5) |
| - Compatible |  | 77 (37) |
| - Incompatible |  | 1 (0.5) |
| Donor steatosis | 162 |  |
| - <10% |  | 148 (91.3) |
| - 10-30% |  | 11 (6.8) |
| - > 30% |  | 3 (1.9) |
| Type of donor | 213 |  |
| - Brain death |  | 211 (99.1) |
| - Cardiac death |  | 2 (0.9) |
| Histology of the explanted liver | 210 |  |
| - Massive or sub-massive necrosis |  | 203 (96.7) |
| Immunosuppression (IS) | | |
| Induction IS | 207 |  |
| -Triple IS (CNI+AZA/MMF+PDN) |  | 147 (71) |
| -Double IS (CNI + PDN) |  | 45 (21.7) |
| -Other |  | 15 (7.3) |
| Basiliximab | 189 | 89 (47.1) |
| Maintenance IS | 190 |  |
| -Mono IS (CNI) |  | 67 (35.3) |
| -Double IS (CNI + AZA/MMF or PDN) |  | 75 (39,5) |
| -Triple IS (ICN+AZA/MMF+PDN) |  | 31 (16.3) |
| -Other |  | 17 (8.9) |
| Early post-LT complications | | |
| ICU days | 212 | 5 (3-10) |
| AKI | 213 | 130 (61) |
| Infection | 211 | 128 (60.7) |
| Respiratory insufficiency | 215 | 48 (22.3) |
| Neurological complications | 213 | 39 (18.3) |
| Bleeding complication | 214 | 41 (19.2) |
| Early graft dysfunction | 214 | 52 (24.3) |
| Biliary complications | 212 | 42 (19.8) |
| CV events | 211 | 7 (3.3) |
| Acute rejection | 211 | 38 (18) |
| -Moderate-severe |  | 27 (79.4) |
| Late post-LT complications | | |
| Chronic rejection | 193 | 17 (8.8) |
| CKD | 188 | 37 (19.7) |
| Recurrence of underlying disease | 190 | 22 (11.6) |
| Biliary complications | 185 | 47 (27.4) |
| AHT | 188 | 57 (30.3) |
| Diabetes | 188 | 32 (17) |
| Dyslipidaemia | 186 | 30(16.1) |
| CV events | 188 | 10 (5.3) |
| De novo tumours | 188 | 14 (7.4) |
| Outcome | | |
| Death | 213 | 58 (27.2) |
| Cause - Infectious | 53 | 22 (41.5) |
| - Liver related |  | 11 (20.8) |
| - Tumour |  | 4 (7.5) |
| - CV |  | 3 (5.7) |
| - Other/mix |  | 13 (24,5) |
| Re-LT | 210 | 27 (12.9) |

Data are given as median (IQR) or number (percentage)

Abbreviations: LT: Liver Transplant, IS: Immunosuppression, CNI: Calcineurin Inhibitor, AZA: Azathioprine, MMF: Mycophenolate mofetil, PDN: Prednisone, ICU: Intensive Care Unit, AKI: Acute Kidney Injury, CV: Cardiovascular, CKD: Chronic Kidney Disease, AHT: Arterial Hypertension

Supplementary Table 3: Gender differences in ALF-LT

| Variable | Men (n=83) | | Women (n=134) | | p-value |
| --- | --- | --- | --- | --- | --- |
|  | n |  | n |  |  |
| Clinical characteristics and management pre-LT | | | | | |
| Age (years) | 83 | 41 (31-56) | 134 | 41.5 (32-52) | 0.999 |
| Race (Caucasian) | 83 | 67 (80.7) | 134 | 114 (85) | 0.402 |
| Body mass index (Kg/m2) | 55 | 26.2 (23.5-29.1) | 97 | 23.8 (20.7-26) | 0.057 |
| Tobacco | 81 | 30 (37) | 131 | 29 (22) | **0.019** |
| Alcohol | 82 | 24 (29.3) | 132 | 8 (6.1) | **<0.001** |
| Drugs | 82 | 12 (14.6) | 132 | 4 (3) | **0.002** |
| AI diseases | 83 | 6 (7.2) | 132 | 25 (18,9) | **0.017** |
| Aetiology | 83 |  | 134 |  | **0.007** |
| - HBV |  | 24 (28.9) |  | 13 (9.7) |  |
| - Other viruses |  | 4 (4.8) |  | 7 (5.2) |  |
| - AI |  | 16 (19.3) |  | 41 (30.6) |  |
| - DILI |  | 16 (19.3) |  | 21 (15.7) |  |
| - Cryptogenic |  | 17 (20.5) |  | 41 (30.6) |  |
| - Other |  | 6 (7.2) |  | 11 (8.2) |  |
| AKI pre-LT | 80 | 44 (52.1) | 133 | 39 (29.3) | **0.001** |
| MELD | 52 | 26 (22-32) | 90 | 23 (18-26) | **<0.001** |
| Creatinine (mg/dL) | 75 | 1.10 (0.8-2.6) | 118 | 0.70 (0.5-0.9) | **<0.001** |
| ALT (UI/L) | 74 | 1126 (474-2441) | 120 | 743 (312-1632) | **0.035** |
| Platelets (x10^3^ /µL) | 73 | 107 (69.6-147) | 120 | 144 (80.4-213.5) | **0.009** |
| King´s College criteria | 77 | 66 (85.7) | 128 | 122 (95.3) | **0.016** |
| Immunosuppression | | | | | |
| Basiliximab prescription | 72 | 41 (57) | 117 | 48 (41) | **0.033** |
| Early post-LT complications, n (%) | | | | | |
| AKI | 82 | 50 (73.1) | 131 | 7 (53.5) | **0.004** |
| Haemorrhage | 83 | 22 (26.5) | 131 | 19 (14.5) | **0.030** |
| Acute rejection | 82 | 9 (11) | 129 | 29 (22.5) | **0.034** |
| Late post-LT complications, n (%) | | | | | |
| Biliary complications |  | 22 (32.4) |  | 25 (21,4) | 0.098 |
| Dyslipidaemia | 69 | 17 (24.6) | 117 | 13 (11.1) | **0.015** |
| Death | 80 | 24 (29.6) | 132 | 34 (25.8) | 0.538 |
| - Death 1yr |  | 16 (20) |  | 21 (15.9) | 0.047 |

Data are given as median (IQR) or number (percentage)

The bold values indicate variables that are statistically significant (p<0.05).

Abbreviations: AI: autoimmune, HBV: Hepatitis B virus, DILI: Drug Induced Liver Injury AKI: Acute Kidney Injury, MELD: Model for End-Stage Liver Disease, ALT: Alanine Aminotransferase.

Supplementary Table 4: Differences in ALF-LT associated with the aetiology

| Variable | AI-DILI (n= 95) | | Other (n= 122) | | p-value |
| --- | --- | --- | --- | --- | --- |
|  | n |  | n |  |  |
| Clinical characteristics and management pre-LT | | | | | |
| Clinical presentation | 95 |  | 120 |  | **0.044** |
| -Hyperacute |  | 23 (24.2) |  | 45 (37.5) |  |
| -Acute |  | 39 (41.1) |  | 49 (40.8) |  |
| -Subacute |  | 33 (34.7) |  | 26 (21.7) |  |
| Time from admission to LT, days | 95 | 6 (3-15) | 122 | 4 (2-10) | **0.043** |
| Diabetes | 95 | 1 (1.1) | 121 | 7 (5.8) | 0.081 |
| AI disease | 94 | 19 (20.2) | 121 | 12 (9.9) | **0.033** |
| AKI pre-LT | 93 | 28 (30.1) | 120 | 55 (45.8) | **0.024** |
| ATB prophylaxis | 83 | 66 (79.5) | 104 | 71 (68.3) | 0.084 |
| MELD - LT Day | 65 | 23.2 (18.5-26.4) | 77 | 26.2 (19-29.9) | 0.093 |
| Donor and surgical characteristics | | | | | |
| Donor steatosis | 77 |  | 85 |  | **0.048** |
| - <10% |  | 66 (85.7) |  | 82 (96.5) |  |
| - 10-30% |  | 11 (14.3) |  | 3 (3.5) |  |
| Immunosuppression | | | | | |
| Induction IS | 91 |  | 116 |  | **0.034** |
| -Triple IS (CNI+AZA/MMF+PDN) |  | 73 (80.2) |  | 74 (63.8) |  |
| -Double IS (CNI + PDN) |  | 13 (14.3) |  | 32 (27.6) |  |
| -Other |  | 5 (5.5) |  | 10 (8.6) |  |
| Early post-LT complications | | | | | |
| AKI | 93 |  | 120 |  | **0.015** |
| -No |  | 36 (38.7) |  | 47 (39.2) |  |
| -Yes (without RRT) |  | 41 (44.1) |  | 34 (28.3) |  |
| -Yes (with RRT) |  | 16 (17.2) |  | 39 (32.5) |  |
| Infection | 93 | 63 (67.7) | 118 | 65 (55.1) | 0.062 |
| -Bacterial |  | 34 (56.7) |  | 50 (79.4) | **0.017** |
| -Fungal |  | 8 (13.3) |  | 6 (9.5) |  |
| -Viral |  | 18 (30) |  | 7 (11.1) |  |
| Bleeding complication | 92 | 12 (11) | 122 | 30(24.6) | **0.020** |
| CV event | 90 | 0 (0) | 121 | 7 (5.8) | **0.021** |
| Late post-LT complications | | | | | |
| De novo tumours | 84 | 3 (3.6) | 104 | 11 (10.6) | **0.034** |
| Death | 92 | 17 (18.5) | 121 | 41 (33.9) | **0.012** |
| - Death 1yr | 92 | 11 (12) | 120 | 26 (21.7) | 0.065 |

Data are given as median (IQR) or number (percentage)

The bold values indicate variables that are statistically significant (p<0.05).

Abbreviations: LT: Liver Transplant, AI: Autoimmune, AKI: Acute Kidney Injury, ATB: Antibiotic, AST: Aspartate Aminotransferase, ALT: Alanine Aminotransferase, MELD: Model for End-Stage Liver Disease, IS: Immunosuppression, CNI: Calcineurin inhibitor, AZA: Azathioprine, MMF: Mycophenolate mofetil, PDN: Prednisone, ICU: Intensive Care Unit, CV: Cardiovascular, CKD: Chronic kidney disease, AHT: Arterial Hypertension

Supplementary figure 1: Patient survival rates over time

The Kaplan-Meier plot illustrates the post-LT survival rates of the 217 ALF patients who underwent LT.

Capsule sentence:

“This multicenter Spanish study describes two-decade trends in liver transplantation for acute liver failure, showing evolving aetiologies, changes in management, improved post-transplant outcomes, and identifying pre-transplant hypertension, acute kidney injury, and hypernatremia as independent predictors of mortality”
