# Supplementary figures and images for: Trends in Liver Transplantation for Acute Liver Failure in a Spanish Multicenter Cohort
Source: Transpl Int. 2025 Dec 16;38:15185. doi: 10.3389/ti.2025.15185 (PMC12752109; doi:10.3389/ti.2025.15185)

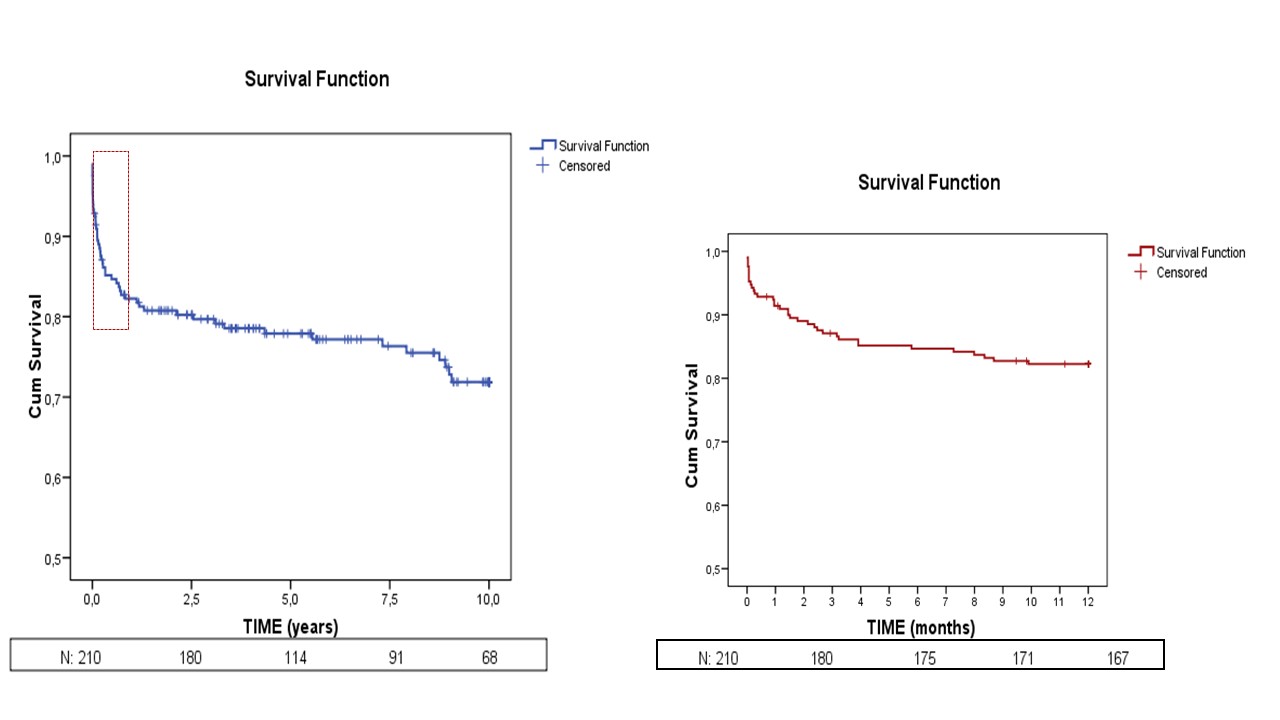

Supplement: Supplementary file 2 [file Image1.jpg]
